# 1679. Successful Control of MSSA Bacteremia Outbreak in Neonatal Intensive Care Unit

**DOI:** 10.1093/ofid/ofad500.1512

**Published:** 2023-11-27

**Authors:** Youngmin Cho, Sujin Choi, Young Hwa Jung, Chang Won Choi, Myoung-jin Shin, Jeong Su Park, Kyoung-Ho Song, Eu Suk Kim, Hong Bin Kim, Hyunju Lee

**Affiliations:** Seoul National University Bundang Hospital, Seongnam-si, Kyonggi-do, Republic of Korea; The Catholic University of Korea, Seoul St. Mary's Hospital, Seoul, Seoul-t'ukpyolsi, Republic of Korea; Seoul National University Bundang Hospital, Seongnam-si, Kyonggi-do, Republic of Korea; Seoul National University Bundang Hospital, Seongnam-si, Kyonggi-do, Republic of Korea; Seoul National University Bundang Hospital, Seongnam-si, Kyonggi-do, Republic of Korea; Department of Laboratory Medicine, Seoul National University Bundang Hospital, Seoul, Seoul-t'ukpyolsi, Republic of Korea; Seoul National University College of Medicine, Seoungnam-si, Kyonggi-do, Republic of Korea; Seoul National University Bundang Hospital, Seongnam-si, Kyonggi-do, Republic of Korea; Seoul National University College of Medicine, Seoungnam-si, Kyonggi-do, Republic of Korea; Seoul National University Bundang Hospital, Seongnam-si, Kyonggi-do, Republic of Korea

## Abstract

**Background:**

*Staphylococcus aureus* outbreaks in the neonatal intensive care units (NICU) have been reported with the great majority due to methicillin-resistant *Staphylococcus aureus*. We describe successful control of an outbreak caused by methicillin-susceptible *S. aureus* (MSSA) in the NICU.

**Methods:**

We report a MSSA bacteremia outbreak which occurred in the NICU at a tertiary level hospital from November 29, 2020 to January 6, 2021. Molecular typing of MSSA isolates were performed including multi-locus sequence typing (ST) and *spa* typing.

**Results:**

A total of 5 MSSA bacteremia cases occurred in 39 days. Outbreak investigation was initiated after the 5^th^ case. Infection control measures, including isolation of cases, contact precaution and universal 0.5% chlorhexidine bathing of all NICU patients, were implemented. Surveillance cultures of patients (n=36) and health care workers (HCW) (n=60) were performed. Excluding bacteremia patients, 13% (4/31) of neonates and 32% (19/60) of HCW were identified as MSSA nasal carriers and targeted decolonization with intranasal mupirocin was performed. After implementation of control measures and decolonization, there were no new cases. Molecular analysis identified two strains [ST15 *spa* type t593 (n=3) and ST101 *spa* type t4171 (n=2)] as the cause of this bacteremia outbreak. Among MSSA nasal carriers, all 4 neonates colonized the ST15 *spa* t593 strain. ST15 *spa* t593 strain was also colonized in one HCW, however antibiotic susceptibility pattern differed. The rest of HCWs colonized many different types of MSSA. All MSSA isolates were negative for Panton-Valentine Leucocidin toxin gene.

**Conclusion:**

Molecular analysis identified two different MSSA strains in this outbreak. MSSA colonization was prevalent among HCWs, however molecular analysis showed variance in distribution. Although outbreak strains were not found in HCWs, one strain was colonized among other neonates, suggesting horizontal transmission and potential of further cases. The outbreak was successfully controlled with reinforcement of precautions, universal chlorhexidine bathing and surveillance cultures with targeted decolonization. Early recognition and prompt implementation of control measures are important for outbreak control in NICU.

**Disclosures:**

**All Authors**: No reported disclosures

